# Load-Bearing Biomedical Applications of Diamond-Like Carbon Coatings - Current Status

**DOI:** 10.2174/1874325000802010043

**Published:** 2008-03-26

**Authors:** Esa Alakoski, Veli-Matti Tiainen, Antti Soininen, Yrjö T Konttinen

**Affiliations:** a ORTON Research Institute, Tenholantie 10, FIN-00280, Helsinki, Finland; b Department of Medicine, Invärtes Medicin, Institute of Clinical Medicine, Biomedicum Helsinki, P.O. Box 700 (Haartmaninkatu 8), FIN-00029 HUS, Finland

**Keywords:** Diamond-like carbon, biomedical applications, load-bearing coatings.

## Abstract

The current status of diamond-like carbon (DLC) coatings for biomedical applications is reviewed with emphasis on load-bearing coatings. Although diamond-like carbon coating materials have been studied for decades, no indisputably successful commercial biomedical applications for high load situations exist today. High internal stress, leading to insufficient adhesion of thick coatings, is the evident reason behind this delay of the break-through of DLC coatings for applications. Excellent adhesion of thick DLC coatings is of utmost importance for load-bearing applications. According to this review superior candidate material for articulating implants is thick and adherent DLC on both sliding surfaces. With the filtered pulsed arc discharge method, all the necessary requirements for the deposition of thick and adherent DLC are fulfilled, provided that the substrate material is selected properly.

## INTRODUCTION

Due to their superior wear and corrosion resistance, DLC coatings have been intensively studied for applications for several decades. DLC coatings have already been applied in automotive parts in racing cars and e.g. in diesel injection systems [1]. A Belgian based multinational company that has facilities in 120 countries; Bekaert is introducing DLC coatings to mainstream automotive industry. Bekaert offers DLC coatings [2] (thicknesses usually between 2 to 4 μm) with the trade name Cavidur. They also offer low friction DLC nanocomposite coatings, with the trade name of Dylyn, with “the best possible combination of anti-stick and wear properties”. Bekaert has announced to have sold over 500 000 automotive valve train components with Dylyn coatings to an unnamed European carmaker [1]. Nissan recently won a Japanese Excellence award from Japanese Ministry of Economy, Trade and Industry (METI) for its hydrogen free DLC coating [3]. Nissan applies its “low friction and highly abrasion resistant DLC coatings” in the valve lifters of their new Skyline and Infiniti G35. However, according to a review on nanocoatings for engine applications by Dahotre and Nayak [4], the major shortcoming limiting the working life of DLC coatings in engine applications is high internal stress and insufficient coating thicknesses.

As diamond is pure carbon and a natural biomaterial also diamond-like carbon coatings can be assumed to be as well biocompatible as hemocompatible. Thus DLC coating should be the superior material for many biomedical applications. There are already few companies that are realising the potential of DLC as a hemocompatible material. Phytis L.D.A. and KIST. J&L Tech MDMI Canada offer DLC coated stents and Cardio Carbon Company Ltd is developing DLC coated artificial heart valve applications. Salahas *et al.* [5] recently published a preliminary clinical study from 245 implanted DLC coated Phytis stents. They conclude that DLC coated stents are associated with high success rates, safety and efficacy, both in hospital and at 6-month follow-up after the intervention. However, there is still apparent lack on commercially applied DLC coatings, especially for load bearing medical applications. Only few unfortunate attempts of commercialisation by small companies in search of quick profits have been made [6]. The main reasons for these failures have been insufficient coating thicknesses and bad materials combinations. Thin DLC coatings cannot be expected to survive when subjected to serious loads, as in artificial hip joints. According to groundbreaking work by Paul [7], the peak loads in human joints can be up to 3.4 and 3.9 times body weight for knee and hip joints respectively.

### Classification

Diamond-like carbon (DLC) is a common term for coatings that have at least some diamond sp^3 ^-bonds in their structure and posses some of the properties of natural diamond. It must be emphasized here that DLC is not a material but a group of materials with a variety of properties. As crystalline DLC coatings are full of grain boundaries, and are prone to crack formation, this review concentrates on amorphous DLC coatings. Amorphous DLC coatings can roughly be divided in to two groups according to their hydrogen content, amorphous hydrogenated carbon coatings (a-C:H) and non-hydrogenated amorphous carbon coatings (a-C). Non-hydrogenated DLC coatings containing high amount of diamond bonds (sp^3^ fraction up to 85%, hardness up to HV 80 GPa) are called tetrahedral amorphous carbon coatings (ta-C).

### Coating Methods

The hydrogen content of the coatings is inherent to the coating method used. Usually a-C:H coatings are deposited using some hydrocarbon gas such as methane or acetylene as a precursor using plasma enhanced chemical vapour deposition (PECVD) methods. A method of producing a-C:H that has been under very much scrutiny during last years has been the plasma immersion ion implantation [8-13] and deposition (PIII + D). In the PIII + D process, target is immersed in plasma. A pulsed high negative voltage is connected to the target, inducing ion implantation. The total surface of the target immersed in the plasma is coated, even without any sample manoeuvring. This technique should be useful for the coating of medical devices with irregular geometries. However, as no evidence of thick coatings deposited with PIII + D can be found in the literature, the usefulness of the method for the deposition of load bearing coatings is still doubtful. According to Chu [14] the plasma energies achieved with this method are low, so that only thin layer of the sample surface is treated. The most common ways of preparing non-hydrogenated DLC coatings are filtered cathodic vacuum arc (FCVA), pulsed laser deposition (PLD) and magnetron sputtering. Of the number of methods of producing DLC coatings only methods with sufficient process yield can be considered for the preparation for practical load-bearing coatings. Unfortunately, often the information on the process yield is excluded from publications.

### Internal Stress

The evident reason for the adhesion problems of DLCs is the high internal stress incorporated into the coating during the violent deposition process. Different approaches have been taken to solve the problem of stress, such as trying to relieve stress e.g. by post deposition thermal annealing [15] or incorporating suitable impurities in the coating during deposition [16-18], using substrate biasing [19, 20] or by ion irradiation [21]. These methods are effective at relieving the stress to at least some degree. The most effective, but somewhat cumbersome way to relieve stress seems to be post deposition thermal annealing. Friedman *et al.* [15] report to have grown a 10 μm thick high quality DLC film with several steps of deposition and annealing. Another, more simple approach of dealing with high internal stresses of DLC coatings is to use suitable substrate materials and plasma acceleration. According to studies conducted by our research group, there are two essential requirements for a substrate material: its hardness must be below HV 3 GPa and it must be able to form carbides [22]. We use the filtered pulsed arc discharge method (FPAD) for the deposition of DLC coatings. We have grown DLC coatings up to thickness of 200 μm [23]. In simulator experiments, our coatings have been proven to reduce the wear of artificial hip joints by a factor of 10^6^ compared to currently available materials [23, 24].

Earlier reviews on biomedical applications of DLC coatings can be found in references [25-27]. Roy and Lee [28] have recently published a somewhat comprehensive review on the earlier work in biomedical applications of DLC coatings. According to them the results of studies done with DLC coatings are controversial. As an example, they use a ten-year clinical follow-up study done with DLC-coated Ti-6Al-4V implants sliding against polyethylene [29]. In the follow-up study the failure rate of DLC-coated femoral head was much higher than alumina femoral head. The failure of DLC in the follow-up study must be due to insufficient adhesion and thin coatings. Thick adherent coatings simply cannot fail against polyethylene. However, thick DLC coatings can lead to increased polyethylene wear. This is due to the fact that thick coatings usually have higher surface roughness. When DLC is sliding against DLC, the contact area gets polished under load and increased wear is not a problem [23]. In some tribological applications sliding pair composed of a hard material and a soft material is successful. However, with load-bearing implants and serum containing fluids as the lubricating medium, the whole approach of trying to improve the wear resistance by increasing the hardness difference of the articulating components has proven itself to be controversial. It must also be noted here that with each step the current commercial UHMWPE acetabular cups release approximately 100 000 wear particles to human body [30]. These wear particles are the main reason behind the aceptic loosening [31] and the eventual revision of the current commercial implants. It is evident that getting rid of the polymer particles and replacing UHMWPE with a wear resistant material, such as DLC, would be extremely beneficial. Thus the focus of research should be shifted to DLC articulating against DLC. That is were the real gains are [23, 24].

## HEMOCOMPATIBILITY OF DLC

Hemocompatibilty of DLC *in vitro* has been intensively studied during recent years. Most hemocompatibility studies have been conducted with a-C:H coatings. The general trend in the results of these studies has been that as most other forms of carbon also DLC has good hemocompatibility. However, no consensus on factors affecting the hemocompatibility has been reached. According to Roy and Lee [28] no consistent relationship can be found between the hemocompatibility and atomic bond structure (sp^3^ -fraction) or the wettability (surface energy) of the surface. Other factors influencing the hemocompatibility may be the interfacial tensions between blood and biomaterial, charge transfer from the protein molecule to the biomaterial surface and the biomaterial surface local texturing [28].

In their recent article Sui *et al.* [32] found that PIII+D deposited a-C:H coating (no thickness given) markedly increased the blood compatibility and the corrosion resistance of NiTi shape metal alloy. According to them the formation of thrombus is correlated with electron transfer from the inactive fibrinogen to the surface of the biomaterial. Insulating materials with high electrical resistivity, such as DLC, inhibit electron transfer and thus decrease the probability of thrombus formation.

Doping with different elements has been proposed for the improvement of the hemocompatibility of DLC. According to Kwok *et al.* [33] the blood compatibility of DLC can be enhanced by doping the film with phosphorus. In *in vitro* platelet adhesion tests they found an optimal concentration of phosphorus that seemed to minimize platelet adhesion. The sample with minimum platelet adhesion had also the smallest interfacial energy with water. The coatings used were very thin (thickness 20-30 nm) and deposited with PIII+D. Andara *et al. *[34] studied the hemocompatibility of DLC-Ag and DLC-Ti composite films and unalloyed DLC films deposited with PLD (their coating thicknesses were approximately 60 nm). In platelet adhesion tests dense networks of fibrin and densely aggregated platelets were observed on the surfaces of DLC-Ag and DLC-Ti coatings. On the other hand unalloyed DLC films did not exhibit fibrin or platelet aggregation during testing, suggesting low tendency to thrombus formation and excellent hemocompatibility. Hasebe *et al*. [35] report that the thrombogenicity of DLC can also be reduced by doping with fluorine. Their coatings were 40-50 nm thick and deposited with radio frequency plasma enhanced CVD. It is believed that surface roughness is a key factor in influencing thrombogenicity [36, 37]. However, Hasebe and co-workers [35] found no significant differences in the platelet-covered area of three F-DLC samples with surface roughness ranging from 4.1 nm to 97 nm. So at least at that roughness range the effect of roughness on the thrombogenicity of F-DLC surface is in doubt.

Maximal adsorbed albumin/fibrinogen ratio is thought to lead to reduced thrombogenesis characteristics of a biomaterial surface. Surfaces that have higher surface energies (lower contact angles) tend to show higher affinity to albumin than surfaces with low surface energies (higher contact angles). However, some authors have also found reduced thrombogenicity on hydrophobic DLC surfaces [14, 38, 39] such as F-DLC. Ma *et al.* [40] tested two a-C:H coatings and one ta-C coating, and do not give the thickness of their coatings. They found that increasing the hydrogen content of the DLC coating leads to lower albumin/fibrinogen ratio. Increasing the hydrogen content also improved macrophage attachment. All DLC coatings in their experiments showed higher albumin/fibrinogen ratio than control materials, silicon and Thermanox^TM^ (chemically resistant polymer treated for enhanced cell attachment and growth). The highest albumin/fibrinogen ratio was on ta-C. Furthermore, ta-C showed higher macrophage attachment than the a-C:H coatings. The DLC coatings induced no toxic effects on the attached macrophages.

## BIOCOMPATIBILITY OF DLC

Most biocompatibility studies concerning DLC are conducted with a-C:H coatings. In fact Roy and Lee do not mention a single biocompatibility study on ta-C in their review [28]. According to Roy and Lee a-C:H films tend to promote the growth and adhesion of cells without inducing any toxicological effect. Studies on the biocompatibility of non-hydrogenated DLC coatings can be found. Some recent hemo- and biocompatibility studies conducted with non-hydrogenated DLC or ta-C are compiled in Table **1**. According to Salguereido *et al.* [41] moderately hydrophilic surfaces induce more favourable cell response than hydrophobic surfaces. In their experiments, MG63 osteoblast-like cells showed poor adhesion on “magnetron sputtered continuous, homogenous and adherent DLC coating” (substrate silicon nitride, no coating thickness given). The surfaces induced no toxic effects on the attached cells. Salguereido and coworkers conclude that DLC coatings are attractive to be used on articulating surfaces of load bearing implants. Bendavid *et al.* [42] have recently studied a-C:H and Si -doped a-C:H films deposited with PACVD method. Silicon is supposed to enhance the antithrombogenicity of a-C:H by inhibiting fibrinogen activation. MG63 osteoblast-like cell attached and grew well on both surfaces. The surfaces induced no toxic effect on the cells. Meunier *et al.* [43] compared the cytocompatibility of a-C:H (thickness 160 nm) and ta-C (thickness 200 nm) coatings deposited with FCVA. The hydrogenated coatings were obtained by introducing methane in the vacuum chamber. These coatings influenced neither the morphology nor the early adhesion behaviour of MC3T3-E1 osteoblast-like cells and indicated optimal surfaces for cell adhesion. No difference between the biological response of the hydrogenated and non-hydrogenated coatings was found. Kinnari *et al.* [44] studied bacterial adhesion (*Stafylococcus aureus*, *Stafylococcus epidermidis*) and the adhesion of human colon adenocarsinoma CACO-2 cells on novel DLC-PTFE-h coating, ta-C, titanium and thermally oxidized silica. The DLC-PTFE-h and ta-C coatings were deposited with FPAD method. The authors found statistically significant reduction in adhesion of stafylococci on DLC-PTFE-h. CACO-2 cells adhered and grew well on all samples. The surfaces induced no cytotoxic effects on the cells.

*In vivo* studies on DLC coated implants are scarce, but those conducted thus far have shown no adverse reactions to DLC coatings. Allen *et al.* [45] tested DLC coatings deposited on CoCr alloy samples in intramuscular implantations of Sprague-Dawley rats and transcortical implantations on skeletally mature ewes. They found no evidence of acute inflammatory reactions or cellular necrosis. Mohanty *et al.* [46] studied DLC coated titanium implanted in skeletal muscle (paravertebral muscles along both sides of the spine) of rabbits. Their coating method was plasma enhanced CVD and coating thicknesses were 1-4 μm. Samples explanted after 1, 3, 6 and 12 months showed no evidence of delamination or release of DLC particles to surrounding tissue. The tissue response to DLC indicated that DLC was biocompatible with skeletal muscle of rabbits. Uzumaki *et al.* [47] implanted DLC coated cylinders made of Ti-13Nb-13Zr into both muscular tissue and femoral condyles of *Rattus Norvegius*. Their coating method was plasma immersion with methane plasma and coating thicknesses were approximately 1 μm. According to a histological analysis the coatings were well tolerated in both types of implantation. La Van *et al.* [48] have published the only *in vivo* study done with ta-C coatings that can be found in the literature. They deposited 400-600 nm thick coatings on both sides of polished silicon wafers using PLD and micromachined the wafers into particles. They also used rapid thermal annealing to reduce the internal stress of the coatings. After six months of implantation they found benign *in vivo* tissue response to ta-C in subcutaneous tissue of SV129 mice. No *in vivo* studies with animals using DLC coated articulating implants was found.

## LOAD-BEARING IMPLANTS

Many papers report studies on DLC coatings sliding against UHMWPE. Such studies have been done with pin-on-disk testers [49, 50] and with hip [49, 51-53] and knee joint simulators [54, 55]. The common trend in these studies is that when the experiments are done in saline solution or water the wear resistance seem to improve somewhat. However, when simulated body fluid, synovial fluid or e.g. calf serum is used no significant improvement compared to uncoated control samples can be seen. In pin-on-disk tests sometimes a protective transfer layer [56, 57] is produced from DLC to the UHMWPE surface. With protein containing lubricants and real loads (the loads used in hip simulators can be up to 3kN [24]) the formation of such layers is evidently inhibited.

Österle *et al.* [58] published recently a pin-on-disk study comparing the wear of several a-C:H coatings (thicknesses 1.7-2.7 μm), ta-C (thickness not given) and other standard hard coatings obtained from commercial coating services. They used an alumina pin as the sliding counterpart in the experiments. According to them a-C:H (nanoindentation hardness similar to alumina) displayed the lowest wear especially with increased hydrogen content. However, after initial tests they excluded ta-C from further experiments due to extensive alumina ball wear. High wear of alumina is hardly surprising as ta-C is significantly harder than alumina. With a ta-C coated ball such wear should not occur.

Only few studies have been made with DLC sliding against DLC. The results of these studies are extremely promising. Reuter *et al.* [59] conducted pin-on-disk studies on five commercially available DLC coatings (thicknesses 1-3 μm) with pin and disk both coated with DLC. The found the lowest wear with the highest quality (ta-C) coating. However, they also found low wear with the two lowest quality (metal doped a-C and metal doped a-C:H) DLC coatings tested. Sheeja *et al.* [60] studied DLC coated Co-Cr-Mo sliding against DLC coated UHMWPE, with a pin-on-disk apparatus (the coating thicknesses were 2 μm and 6 μm, respectively). They used a FCVA system with substrate biasing for the deposition of the DLC coatings. The hardness of the DLC coating was relatively low, only HV 28 GPa (hardness of ta-C can be up to HV 80 GPa). They found that DLC coating on both sliding surfaces decreased the wear of the UHMWPE pin by a factor of 10^4^ compared to uncoated UHMWPE pin sliding against Co-Cr-Mo disk. The test was done in simulated human serum. Sheeja and co-workers concluded that adhesive and thick DLC coating on both sliding surfaces of the Co-Cr-Mo/UHMWPE implants could be a choice to prolong the life of implants. They admit that much more experiments in a hip simulator, with real loads and a suitable lubricant medium are needed. However, when considering real loads the thicknesses of their coatings still seem insufficient. Also the violent coating process may have adverse effects on the UHMWPE/DLC interface.

Our research group has extensively studied DLC coatings since the late 80’s [22-24, 44, 49, 61-74]. During last 10 years our focus of research has been on ta-C articulating against ta-C. In our studies the ta-C/ta-C sliding interface has shown its superiority in pin-on-disk experiments [23, 49] and in experiments conducted with a custom-made hip joint simulator [23, 72]. In experiments with an accredited Shore &Western hip joint simulator with bovine serum as a lubricating medium, a 60 μm thick ta-C coating on both interfaces reduced the wear of hip implant by a factor of 10^6^ compared to commercially available materials [24]. It must also be emphasized that this is an estimation of the maximum wear, as wear in the experiments was near the detection limit. Excellent adhesion is achieved in the system by using high plasma energies in the beginning of the deposition process, so that sufficient interfacial mixing can be achieved [22, 61, 64]. Also, as mentioned earlier the substrate material must be a carbide former and its hardness must be below HV 3 GPa. Unfortunately proper substrate materials usually have a native oxide layer on their surface. This surface oxide has to be removed e.g. by argon sputtering if adhesive films are to be deposited. According to a recent unpublished study by Tiainen *et al.* high energy carbon ions are able reduce the oxide layer. In Fig. (**1**) the calculated range of 1 keV carbon ions in silicon is seen. With these energies, easily achievable in the system, the carbon ions are able to reduce away the whole, approximately 1.5 nanometers thick, oxide layer [75] and induce a 15 nm thick interfacial mixing layer, leading to excellent adhesion.

Recently T.J. Joyce [76] published an *ex-vivo* case study on a metatarsophalangeal (MTP) implant with DLC coating on both its articulating faces. After four years of implantation the DLC coating had been completely removed from the entire face of the phalangeal component and from the most of the face of the metatarsal component. The main failure mechanism was thought to be corrosion at the coating substrate interface. MTP implant used in the study was made from cobalt chrome alloy and the coating thickness was approximately 350 nm. No information on the deposition method is given. With thin coatings there is a high probability of pinholes that provide corrosion paths through the coating. According our earlier results [23] DLC coatings should be at least 1 μm thick to significantly reduce the pinhole effects. Of course a 1 μm DLC coating is still very thin. To withstand the forces present in a human joint the coating should be at least an order magnitude thicker. Furthermore, even in theory the cobalt chrome alloy substrate is simply too hard for the deposition of thick DLC coatings [22] (without suitable intermediate layers). Consequently the thin coating failed under load.

As noted by Hauert [6] in his excellent and well known review article there have been very few attempts at bringing DLC commercially available in load bearing implants. A French company M.I.L SA offered DLC coated titanium shoulder-joint balls and ankle joints with both the talar and tibial components made from nitrided AISIZ5 CNMD 21 steel and coated with DLC. However, their DLC coated implants were evidently not a major success story, as the company went bankrupt and no studies on their implants can be found in the scientific literature. After bankruptcy, the founders of M.I.L SA founded a new company called I.Ceram (Limoges, France), offering orthopaedic implants. Despite several request for references to their work on DLC coatings, no additional information was received from I.Ceram.

A sad example of a failure is the Swiss company Implant design AG that sold the so-called “Diamond Rota Gliding” knee implant [6]. They brought to market an insufficiently tested implant without required marketing licences (they used e.g. the European CE -marking on their products without having obtained licence to do so) [77]. The tibial component of their implant was coated with DLC and the femoral component was made of UHMWPE. After some of their implants failed in a very short time due partial coating delamination and high wear, the Swiss Federal Office of Public Health banned the implant. The company went bankrupt and public lawsuits (Swiss Channel SF TV news 4.10.2001) were raised against the operating surgeons.

## DISCUSSION

The survival of the DLC coating under load is proportional to the square of the coating thickness [78]. This makes the thickness of the coating of the utmost importance, when depositing coatings for load bearing applications. Thick coatings make also the probability of existence of pinholes providing corrosion paths through the coating to the substrate very low. Such corrosion paths can lead to galvanic effects, delamination and eventual gradual or catastrophic failure of the coating. Furthermore, it takes a long time for the lubricating fluid to diffuse through microscopic pinholes. Moderately thick coating might thus be successful in simulator experiments but fail *in vivo*. Thus, accelerated simulator experiments should also be done only with thick coatings. Unfortunately, often experiments are done with very thin < 1μm coatings, and sometime the thickness information is entirely omitted from publications. This is probably due to the inability of the experimentalists to deposit thick well-adhesive coatings. Of course biocompatibility studies can be done with thin coatings. However, under severe loads such thin coatings will most probably fail.

Thicker coatings usually mean also higher surface roughness. Higher surface roughness usually leads to higher wear. However, with ta-C coatings after the initial polishing of the surface the wear of coating is practically non-existent. In an experiment, with a custom-made simulator, a hip joint manufactured of AISI316L with a ta-C coating of 60 μm withstood 100 000 walking cycles under a weight of 1300 kg without any wear or damage [23]. As DLC is a hard ceramic material, it is also very important that the fitting of the implant is done carefully and the tolerances are precise. In the aforementioned experiment the polished contact area of the implant was only a few square millimetres, due to poorly done fitting of the implant. Without excellent adhesion the high peak loads would have destroyed the coating. Even with this high loads audible squeaking sometimes observed in some ceramic-on-ceramic implants [79, 80] was not present.

The usual approach nowadays seems to be to select a substrate biomaterial for testing and then try to coat it with DLC. Usually the selection is done on the basis of the biological properties and the biocompatibility of substrate material, or simply by selecting a material that has been used before in biomedical applications. This approach is doomed to fail, as some biomaterials are simply impossible to coat at least with thick coatings necessary for load-bearing applications. Typical example of this is the work which compared the wear resistance of DLC coatings deposited on implant alloys CoCrMo and Ti-6Al-4V with a ring on disk wear tester [81]. It was found that Co-Cr-Mo is the superior of the two of these. However, the thickest coatings tested were only 1.2 μm thick. According to our experience [22] both these materials are too hard for the deposition of thick coatings. Substrate materials used and maximum DLC coating thicknesses achieved on them in recent studies are shown in Table **2**.

The more practical way to proceed would be to select a material that is reasonably biocompatible and that can be coated with thick coatings. One such material is surgical steel AISI316L. Thick protective coatings will prevent the adverse effects from the perhaps less biocompatible components of the substrate material.

An appealing way of realising the potential of DLC would be to use DLC on both articulating surfaces of the new metal on metal resurfacing Birmingham implants. Resurfacing implants are used with younger and more active patients. The procedure conserves patients own bone stock and the shape of the femoral head of the implant mimics the shape of the human natural femoral head more closely. Also, as the resurfacing implant replicates the size of the ball and socket i.e. the anatomy of the human hip better than the conventional hip replacement the risk of dislocation after the surgery is smaller [82].

Such ill-advised attempts at bringing to market poorly tested half completed products, as the “Diamond rota gliding” knee, are very unfortunate. They contribute to prejudices against DLC coatings already existing in the medical implant industry and may thus lead to further postponement of viable coating applications. At the same time every year tens of thousands of more patients worldwide will begin to suffer from the consequences of implant wear and corrosion.

## CONCLUSIONS

Although many papers reporting studies on DLC sliding against UHMWPE have been published, this approach in improving articulating implants with DLC seems to be a dead-end. No significant improvements to current materials have been reported in simulator studies conducted with real loads and serum containing lubricating fluids. The superior way of realising the potential of DLC in load bearing implants e.g. shoulder, hip, knee and ankle implants is to use thick DLC coatings on both articulating surfaces. For the purpose of depositing thick high quality DLC coatings, only one method with a proven track record can be found in the literature, the filtered pulsed arc discharge method. Excellent simulator results on DLC coated artificial hip joints have been published already in the late 90’s [23]. It is an extremely unfortunate fact that these results have not awakened much interest in the implant industry. It seems that large implant companies have no comprehension on the importance of DLC coated implants. Instead, hundreds of thousands of patients will continue suffer from the effects of implant corrosion and wear. It would be of utmost importance to obtain basic well-tested implant models for testing. The models should be manufactured from materials, such as AISI316L, that can be coated with thick DLC coatings. Unfortunately, for small research groups with limited resources, obtaining such implant models is extremely difficult. For a large implant company, with the necessary interest, there should be no problems in this.

## Figures and Tables

**Fig. (1):**
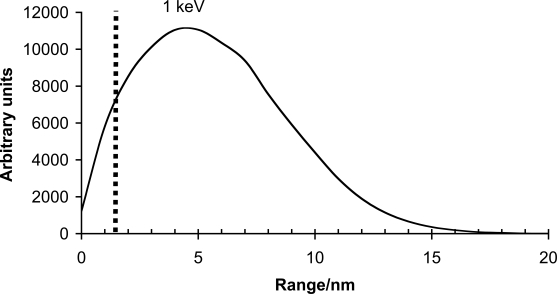
Calculated 1 keV C+ ion range in silicon. The carbon ions reach well beyond the thickness of the native oxide layer shown with the dashed line in the graph. The simulations were conducted with TRIM (SRIM-2003.26) using 10^5^ ions.

**Table 1:** Recent Biocompatibility and Hemocompatibility Studies Conducted with Non-hydrogenated DLC/ta-C

Study	Result, Coating Type, Deposition Method
Static bacterial and CACO-2 cell adhesion, *in vitro* Kinnari *et al.* [44]	Statistically significant reduction in adhesion of stafylococci in DLC-PTFE-h. CACO-2 cells adhered and proliferated well. No cytotoxic effects on the cells, ta-C, FPAD
Albumin/fibrinogen adsorption, macrophage attachment, *in vitro,* Ma *et al. *[40]	High albumin/fibrinogen ratio, higher macrophage attachment than controls, non toxic to macrophages, ta-C, FCVA
MG63 osteoblast-like cell adhesion *in vitro,* Salguereido *et al.* [41]	Poor adhesion of the cells, however adherent cells displayed normal morphology on the surface, non-H DLC, magnetron sputtering
Platelet adhesion test *in vitro,* Andara *et al*. [34]	Unalloyed DLC films did not exhibit fibrin or platelet aggregation during platelet rich plasma testing, ta-C, PLD
Cytocompatibility with MC3T-E1 osteoblast-like cells Meunier *et al*. [43]	No influence on the morphology or early adhesion behaviour of cells, ta-C, commercial FCVA
*In vivo* test, subcutaneous tissue of SV129 mice (6 mths), Lavann *et al*. [48]	Mild tissue reactions constrained to the area of injection or implantation, ta-C, PLD

**Table 2:** Substrate Materials, Maximum Deposition Thicknesses and Deposition Methods Used in Recent Biomedical Application Studies with DLC Coatings

Substrate Material	Thickness	Dep. Method	Ref.
Co-Cr-Mo, implant alloy	2 µm	FCVA	Sheeja *et al*. [60]
Ni-Ti, shape memory alloy	0.6 µm	PIII + D	Liu *et al.* [9]
Silicon, test substrate	2 µm	PIII + D	Yokota *et al.* [8]
Silicon nitride (Si_3_N_4_), hard ceramic	None given	Mag. sputtering	Salguereido *et al*. [41]
Silica glass, test substrate	2 µm	PIII + D	Yokota *et al. *[8]
Ti-13Nb-13Zr, proposed implant alloy	1 µm	PIII + D	Uzumaki *et al*. [47]
Ti-6Al-4V, implant alloy	2.7 µm	Mag. sputtering	Österle *et al*. [58]
AISI316L, surgical steel	200 µm	FPAD	Anttila *et al.* [23]
Cr-Mn-N, steel (P2000)20 % strain hardened	2 µm	PLD	Reuter *et al.* [59]
UHMWPE Implant polymer	6 µm	FCVA	Sheeja *et al*. [60]
